# Image segmentation algorithm based on improved YOLOv8 model and its application in underground coal and gangue recognition

**DOI:** 10.1371/journal.pone.0321249

**Published:** 2025-05-09

**Authors:** Lei Zhu, Wenzhe Gu, Chengyong Liu, Beiyan Zhang, Wentao Liu, Chaofeng Yuan

**Affiliations:** 1 China Coal Energy Research Institute Co., L td., Xi’an, Shaanxi, China; 2 China Coal Xi’an Design Engineering Co., L td., Xi’an, Shaanxi, China; Universidad de Almeria, SPAIN

## Abstract

Coal and gangue recognition technology is one of the key technologies in the intelligent construction of coal mines. With the deepening of the research, only the coal and gangue recognition, the pixel segmentation of the coal gangue image is needed. Aiming at the gangue segmentation algorithm with low accuracy, easy to miss detection, wrong detection and large amount of detection data, slow detection speed and other problems. A coal gangue segmentation model based on improved YOLOv8 is proposed to achieve fast and accurate recognition of coal gangue images, and the overall computational volume of the model is not large, which has achieved better application results. Using the YOLOv8 model as the base model, the standard convolutional modules in the first, second & third C2f modules were replaced with depth separable convolution (DSC) modules in the YOLOv8 model backbone network, reducing the overall computational effort of the model. Adding the CBAM module before the second convolution of the up-sampling module and down-sampling stage in the model neck network improves the differentiation of the model for gangue and enhances the recognition accuracy. The original dataset was expanded from 1980 to 11,265 sheets using data expansion techniques and some hyperparameters were adjusted. Results show that the improved YOLOv8 model has an accuracy (Precision) of 95.67%, a recall (Recall) of 95.74%, a transmitted frames per second (FPS) of 32.11 frames/s, and a mean average precision (mAP) of 96.88%, which is an improvement of 5.6% in accuracy, 7.12% in recall, and the mean average precision (mAP) is improved by 4.65% and FPS is improved by 8.83 frames/s. By comparing with YOLOv3, YOLOv5, YOLOv7, and YOLOv8 models, the improved model is optimal in terms of accuracy and speed. Finally, the model is successfully applied to underground coal gangue image segmentation through transfer learning, and the effect of coal gangue image segmentation is good, which verifies the re-liability of the algorithm.

## 1 . Introduction

Intelligent mining is an important guarantee for safe, efficient and green mining. In the process of coal mining [1–4], how to reduce the gangue rate has been a major mine production problem, specifically in the shaft gangue sorting and underground coal caving opening and closing, gangue recognition technology is the core technology of coal gangue sorting and caving opening and closing timing, the common recognition methods are mainly artificial recognition, natural γ-ray recognition [5,6], vibration signal recognition [7,8] and computer vision recognition [9,10]. Artificial recognition is affected by subjective factors, and it is difficult to guarantee the recognition accuracy; natural γ-ray recognition needs to be carried out under the high level of radioactive conditions, and the recognition accuracy is limited under the condition of low level of radioactivity of the coal and gangue; vibration signal recognition is susceptible to the signal interference of mechanical noise, personnel activities, etc. Computer vision can choose different algorithms according to different light and pixel conditions [11,12], and has wide compatibility for coal and gangue recognition [13,14], therefore, the key support for coal and gangue recognition of computer vision technology.

In recent years, with the development of machine vision technology in the field of coal and gangue recognition, domestic and foreign researchers and scholars have conducted a lot of research in this field and achieved many useful conclusions. There are two main classes of algorithms for image detection based on deep learning. One is the target detection algorithm represented by Faster R-CNN [15]. The other is the target detection algorithm represented by Yolo series. The YOLO series of models has been attracting the attention of the majority of scientific researchers in the field of image recognition because of its high accuracy, fast computation, etc. [16–18], and it has been updated to YOLOv11 from YOLOv1.The series of models have been used in the field of coal and gangue recognition. The series of models have good performance in downhole personnel behavior recognition and coal and gangue recognition, but the initial release of the open-source model for the recognition of specific targets lack of relevance, there is a certain recognition error, in the research usually according to the use of the back-ground of the network to improve the adaptability of the model to the application.

Pu [19] et al. used convolutional neural network (CNN) to identify coal and gangue images, based on the findings of the study to guide the separation of coal and gangue and proposed a workflow for CNN image recognition and a method to update the model parameters. Zhang [20] et al. added the Flip-Mosaic algorithm to the improved YOLOv5 algorithm for monitoring vehicle video streams, which further improved the vehicle detection accuracy. Wang [21] et al. improves the network structure on the basis of the YOLOv5s deep learning network, introduces Slim-neck, the SimAM attention mechanism and decoupled head, and proposes the decoupled Head-GSConv-VoV-GSCSP-YOLOv5s-SimAM (DGSV-YOLOv5sSA) network. Cao [22] et al. proposed an improved lightweight target detection model for Yolov5s, which uses the visual activation function FReLU to adaptively extract image context information, modifies the neck structure of the network to achieve the detection capability for small samples, and replaces some of the convolutional blocks in the original network with DWC and GSC modules, respectively. The ECA module is added to the backbone net-work to further improve the feature extraction capability of the model. Shang [23,24] et al. optimized the feature extraction structure by introducing a part of the lightweight net-work GhostNet in the backbone network, effectively reduced the computational parameters of the model, optimized the computational capability of the model, and introduced a SimAM attention mechanism module in the head of the model, which enhanced the learning ability of coal and gangue features. Yang [25] et al. proposed a coal gangue recognition method based on XBS-YOLOv5s, which improves the model’s ability to extract coal and gangue features by fusing the SimAM attention mechanism, BiFPN network and XIoU loss function in YOLOv5s.

The above research has achieved remarkable results in the field of coal and gangue recognition, but the recognition method is to mark coal and gangue with different color boxes in the picture, and this method can not accurately get the percentage of coal and gangue in the photo, this paper expands the image recognition on the basis of previous research, by adding depthwise separable convolution (DSC) to the back-bone network, adding the module of attention mechanism (CBAM) to the model neck network, and improving the YOLOv8 model. The coal and gangue image segmentation is achieved, and the pixel occupancy ratio of coal and gangue can be accurately obtained by image segmentation, which is a big improvement over the conventional coal and gangue recognition research.

The rest of the paper is organized as follows. Section 2 describes in detail the introduction of the YOLOv8 model and shows how it can be improved on the structure of the original YOLOv8 model; in Section 3, the source of the coal and gangue recognition image dataset is shown; in Section 4, the computational results of the present model are shown, which are compared and analyzed with the common recognition models; and finally, the conclusions of the full paper are summarized.

## 2. Improvements to the YOLOv8 model

### 2.1 Introduction of YOLOv8 model

YOLOv8 model is a YOLO series of detection framework proposed in 2023, YOLOv8 model has improved model structure, accuracy, training strategy, feature enhancement and deployment optimization relative to YOLOv5 model, YOLOv8 model has a fast inference speed while maintaining high detection accuracy, and relative to other complex target detection models, the YOLOv8 model has a smaller model size and memory footprint [26–28]. An end-to-end training method is used to perform target detection directly from the original image pixel level, without the need for complex preprocessing or multi-stage processes. The network structure is clear and concise, easy to understand and modify, and developers are able to quickly deploy and adapt the model in different application scenarios to meet specific needs.

**Fig 1:**
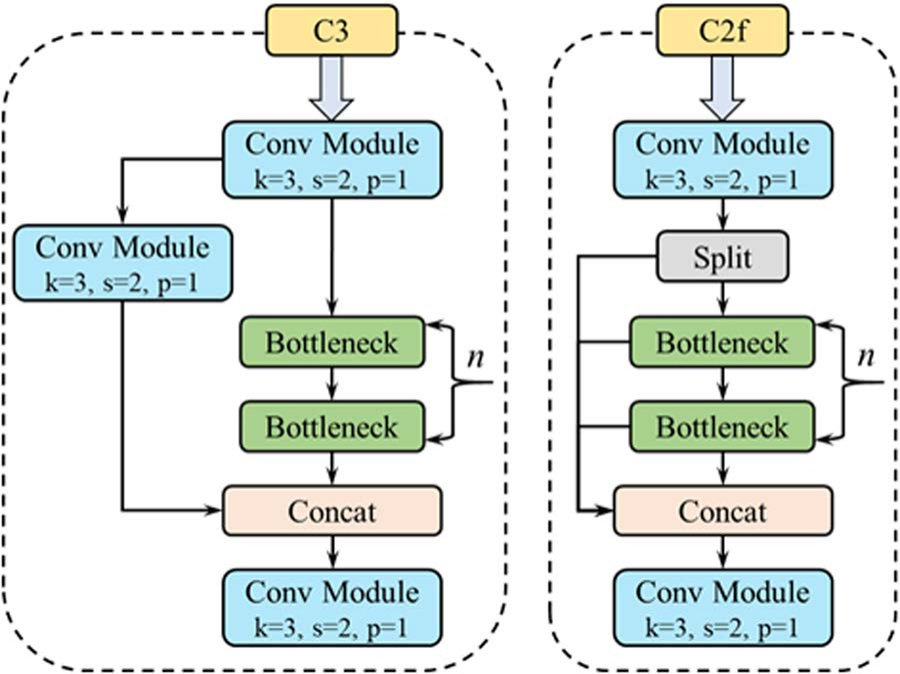
Schematic diagram of C3 and C2f module structure.

The backbone network of YOLOv8 model adopts C2f module instead of C3 module, and the C2f module adds more branches, which refer to the viewpoints of C3 module and ELAN, so that the YOLOv8 model obtains more gradient flow information. Fig 1 shows the structure of C3 module on the left side and the structure of C2f module on the right side.

The YOLOv8 model detection head is a “decoupled head” structure, i.e., classification and localization are extracted from two parallel branches respectively, and the latter is completed by 1 × 1 convolution.

The label classification strategy of YOLOv8 model adopts TOOD dynamic label allocation strategy, and the main losses are category loss, location loss, and regression loss in the form of CIoU_Loss and DFL_Loss.

The category loss is calculated as equation (1):


$$VFL(p,q)=\left\{ \begin{array}{cc} & -q(q\log(p)+(1-q)\log(1-p)),q>0\\ & -\alpha p^{\gamma }\log(1-p),q=0 \end{array}\right.$$ (1)

Where: p is the predicted probability of belonging to a label between 0 and 1; q is the IoU value of the predicted frame and the real frame when the target is a positive sample, and q is 0 when it is a negative sample.

The regression loss CIoU_Loss is the intersection and concurrency ratio between the predicted frame and the real frame, and DFL_Loss mainly allows the model to quickly focus on the distribution of the location that is close to the target location, CIoU_Loss is defined as equation (2), and DFL_Loss is defined as equation (3):


$$CIoU=1-IoU+\frac{\rho^{2}(b,b^{gt})}{c^{2}}+\alpha v$$ (2)

$$DFL(S_{i},S_{i+1})=-((y_{i+1}-y)\log(S_{i})+(y-y_{i})\log(S_{i+1}))$$ (3)

Where: IoU is the intersection ratio between the prediction frame and the actual frame; ρ is the distance of the actual detection center from the prediction center; c is the diagonal length of the detection frame; α is the weighting coefficient; v is the width-to-height ratio of the prediction frame; y is the target value; and S is the cross-entropy.

### 2.2 Depthwise separable convolution

Depthwise separable convolution decomposes standard convolution into two simpler operations: channel-by-channel convolution (Depthwise Convolution) and point-by-point convolution (Pointwise Convolution). This module significantly reduces the overall amount of computation and number of parameters in the model, thus improving the computational efficiency of the model.

**Fig 2:**
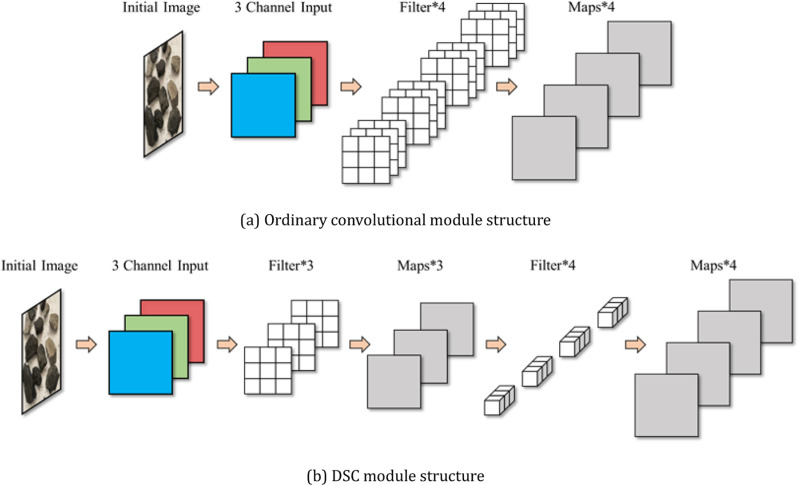
Comparison of convolutional module structures. (a) Ordinary convolutional module structure. (b) DSC module structure.

Depthwise separable convolution reduces the number of input channels and thus effectively reduces the parameters required for the convolutional layers, while running faster and less computationally intensive than traditional convolution, making it easier to implement and deploy on different platforms. The ability to streamline the computational model leads to high accuracy on smaller devices. Fig 2 shows the comparison between the structure of ordinary convolutional module and the structure of deeply separable convolutional module.

The ratio of the number of DSC parameters to the number of ordinary convolutional parameters is shown in equation (4):


$$\frac{D_{K}\;\;\times \;\;D_{K}\;\;\times \;\;M+M\;\;\times \;\;N}{D_{K}\;\;\times \;\;D_{K}\;\;\times \;\;M\;\;\times \;\;N}=\frac{1}{N}+\frac{1}{D_{K}^{2}}$$ (4)

The ratio of DSC computation to ordinary convolutional computation is shown in equation (5):


$$\frac{D_{K}\;\;\times \;\;D_{K}\;\;\times \;\;M\;\;\times \;\;D_{F}\;\;\times \;\;D_{F}+M\;\;\times \;\;N\;\;\times \;\;D_{F}\;\;\times \;\;D_{F}}{D_{K}\;\;\times \;\;D_{K}\;\;\times \;\;M\;\;\times \;\;N\;\;\times \;\;D_{F}\;\;\times \;\;D_{F}}=\frac{1}{N}+\frac{1}{D_{K}^{2}}$$ (5)

Where: *M* is the number of depth-by-depth convolution kernels; *N* is the number of point-by-point convolution kernels.

Taking the common 3 × 3 convolution kernel as an example, the DSC is about 1/9 of the ordinary convolution in terms of the number of parameters and computation, which greatly improves the computational efficiency.

### 2.3 Attention mechanism

The common attention mechanism modules are SE (Squeeze-and-Excitation), CBAM (Convolutional Block Attention Module), ECA (Efficient Channel Attention), CA (Channel Attention), etc.. In deep learning applications, SE is suitable for high-channel-count scenarios, CBAM provides spatial and channel attention, ECA improves performance while maintaining efficiency, and CA takes spatial dimensions into account, and the choice needs to be based on the application scenario to weigh the computational cost and effectiveness. CBAM improves the model performance through adaptive feature recalibration and dual-attention mechanisms, with high computational efficiency, and the authors’ previous findings also confirmed that CBAM is more suitable as an attention mechanism module for coal gangue image recognition.

**Fig 3:**
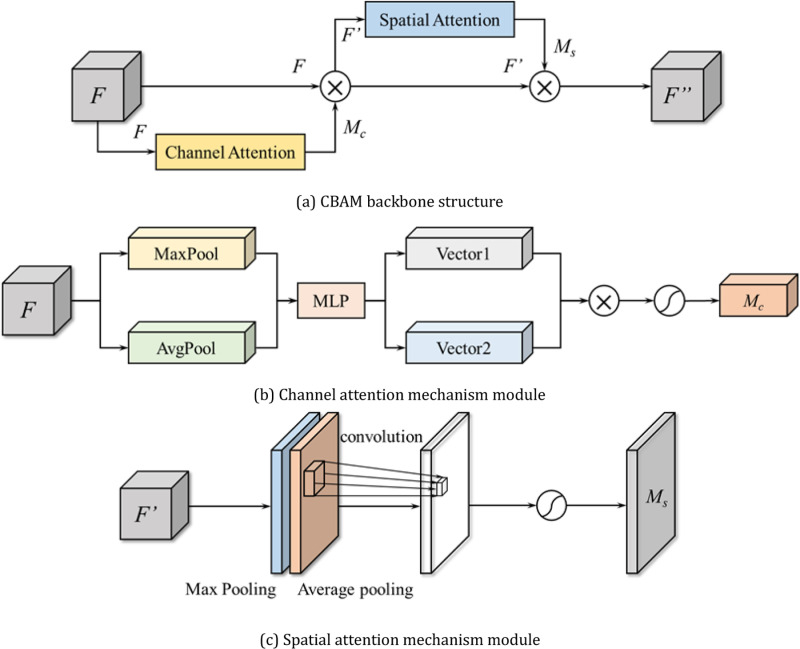
Structure of the CBAM module. (a) CBAM backbone structure. (b) Channel attention mechanism module. (c) Spatial attention mechanism module.

CBAM attention mechanism consists of channel attention mechanism (Channel) and spatial attention mechanism (Spatial), which introduces two analysis dimensions, spatial attention and channel attention, to achieve the sequential attention structure from channel to space. Spatial attention allows the neural network to pay more attention to the pixel regions in the image that play a decisive role in classification and ignore irrelevant regions, and Channel attention is used to deal with the allocation relationship of the feature map channels, and the structure of CBAM with each channel is shown in Fig 3.

### 2.4 Improved YOLOv8 model structure

In this paper, the depthwise separable convolution module (DSC) and the attention module (CBAM) are added into the backbone network of YOLOv8, which can make the whole algorithm accuracy and computational efficiency significantly im-proved, because: (1) when the amount of data in the coal gangue image is too large, the depthwise separable convolution is more robust and computationally efficient in sampling, which is difficult to be achieved by the ordinary convolution; (2) the size of coal gangue lumps in the image is relatively small, and the shape difference is large, which makes it difficult to achieve accurate detection of small targets. smaller, and the shape difference is larger, ordinary convolution is difficult to achieve accurate detection of small targets, and it is easy to wrong detection, omission, which leads to the performance of the model; (3) CBAM module has a smaller amount of computation, and it is more sensitive to the important information of the image, and it has a high computational efficiency, and it has a good adaptability to the detection task.

For this reason, this study chooses to replace part of the ordinary convolution module with depthwise separable convolution module in the backbone network of YOLOv8, and add the attention mechanism module in the neck of the network structure, which can further improve the computational efficiency and robustness of the model, enhance the accuracy of small target detection, and make the prediction of the detection head of the “decoupled head” structure more stable. The “decoupled head” structure makes the prediction of the detection head more stable.

**Fig 4:**
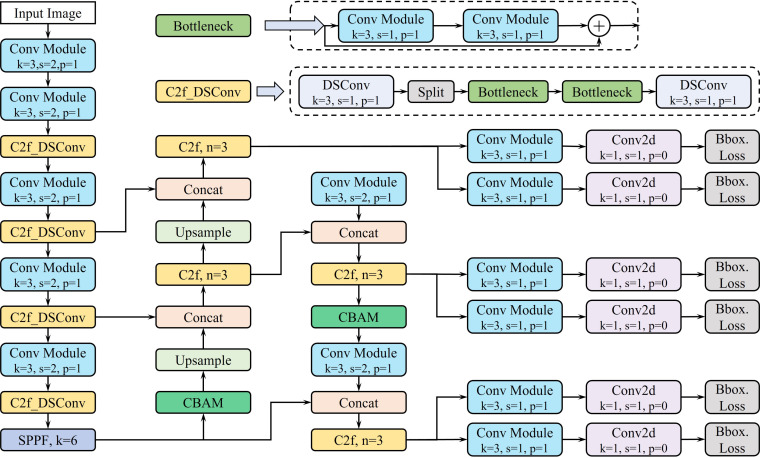
Improved YOLOv8 network structure diagram.

The standard convolution of the first, second and third C2f modules in the back-bone network of YOLOv8 is replaced with depthwise separable convolution to reduce the computational volume of the model, and the CBAM module is added in front of the Upsample Module in the up-sampling stage of the neck and in front of the second Conv Module in the down-sampling stage to increase the model’s focus on the small targets, thus improving the overall performance of the model. The network structure of the improved YOLOv8 model is shown in Fig 4.

## 3. Coal and gangue recognition model dataset construction

### 3.1 Coal gangue image acquisition

The gangue used in this paper is the real gangue, which is manually placed ac-cording to a certain randomness and then photographed to collect images with image resolution of 640 × 640 of the combination of different gangue sizes and morphologies, and a total of 1980 original images are obtained.

**Fig 5:**
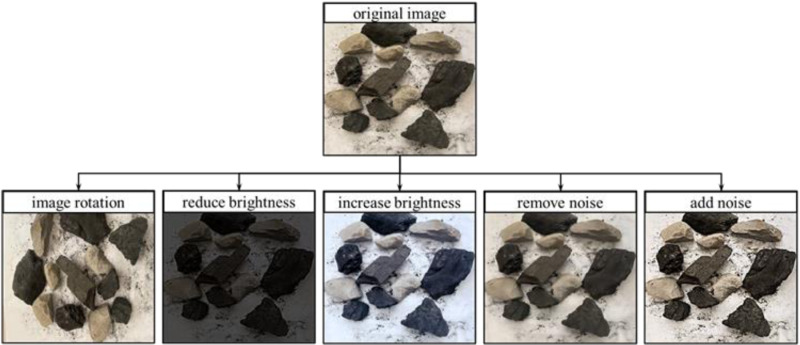
Comparison of the effect of different image expansion methods.

Sample training with less data is prone to randomness, in order to restore the conditions of multi-factors influence in the field to a greater extent and improve the generalisation of the algorithm, the original image is rotated by image rotation (randomly rotated 90, -90 degrees), decreasing the luminance (randomly decreased by 0–50%), increasing the luminance by 50% (randomly increased by 0–50%), removing the noise (in order to simplify the calculations, the arithmetic mean filter is selected for simple de noise), adding noise (randomly generating black and white pixels on the image, pepper and salt noise = pepper noise (value 0, black) + salt noise (value 255, white)) for dataset expansion, and ultimately get 11,265 pieces of gangue samples of the image, the image rendering effect of the different ways of expanding the image and the original image comparison is shown in Fig 5.

### 3.2 Image annotation and classification

**Fig 6:**
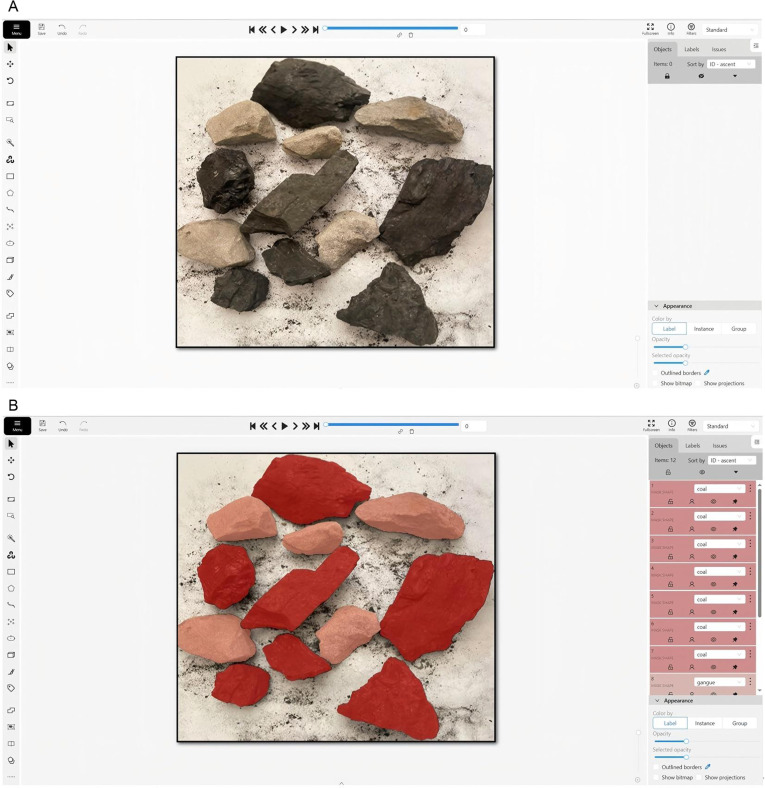
Image annotation results. (a) Image import. (b) Image segmentation annotation.

The coal and gangue image dataset is divided into training set, test set and validation set by random sampling in the ratio of 8:1:1. Using the ‘Segment’ function in the CVAT annotation tool to annotate the coal and gangue images, the imported original image and the labelled result are shown in Fig 6(a) and Fig 6(b), the annotated image contains 68,475 coal labels and 47,301 gangue labels.

## 4. Experimental testing of coal and gangue recognition

### 4.1 Experimental environment and hyperparameters

In this paper, Pytorch deep learning framework is used for model training, and the experimental hardware equipment is Intel(R) Core (TM) i9-10850K CPU @ 3.60GHz processor, 64GB RAM, and NVIDIA GeForce RTX 3070 graphics card with 16GB video memory size. The software environment is Windows 11 operating system, python3.8, pytorch1.9.0 deep learning framework, and CUDA version 11.1.

The hyper-parameters are set as follows: the number of training rounds is 301, the learning rate is 0.01, the cosine annealing algorithm is selected for the learning rate adjustment strategy, the optimizer is chosen to be the Adam optimizer, the batch size is 32, the pixels of the input image are 640 × 640, the stochastic gradient descent method is used for the optimization of the model, the number of iterations epoch is 50, the momentum (momentum coefficient) is 0.937, the decay (weight decay coefficient) is 0.0005 [29–31].

### 4.2 Indicators for model evaluation

In this paper, detection precision (Precision), recall rate (Recall), average precision mean (Map), and frames per second transmission (FPS) are used as the evaluation indexes of the improved YOLOv8 model, and the formulas of the related indexes are shown in Eqs. (6) to (10).


$$Recall=\frac{TP}{TP+FN}$$ (6)

$$Precision=\frac{TP}{TP+FP}$$ (7)

$$AP=\sum_{i=1}^{n}\left(Ai\times Pi\right)\sum_{i=1}^{n}Pi$$ (8)

$$mAP=\frac{\sum {\mathrm{\backslash nolimits}}_{i=1}^{k}AP_{i}}{k}$$ (9)

$$FPS=\frac{1000}{T}$$ (10)

In the above equation:

*TP* is the number of true targets classified as true by the classifier;

*FP* is the number of false targets classified as true by the classifier;

*FN* is the number of true targets classified as false by the classifier;

*TN* is the number of false targets classified as false by the classifier;

*A*_*i*_ is the number of True Positive at the ith IoU (Intersection over Union) value;

*p*_*i*_ is the prediction accuracy at the ith IoU value;

The mAP chosen for this paper is mAP@0.5 (inter-parallel ratio of 0.5).

### 4.3 Experimental results and analysis

**Fig 7:**
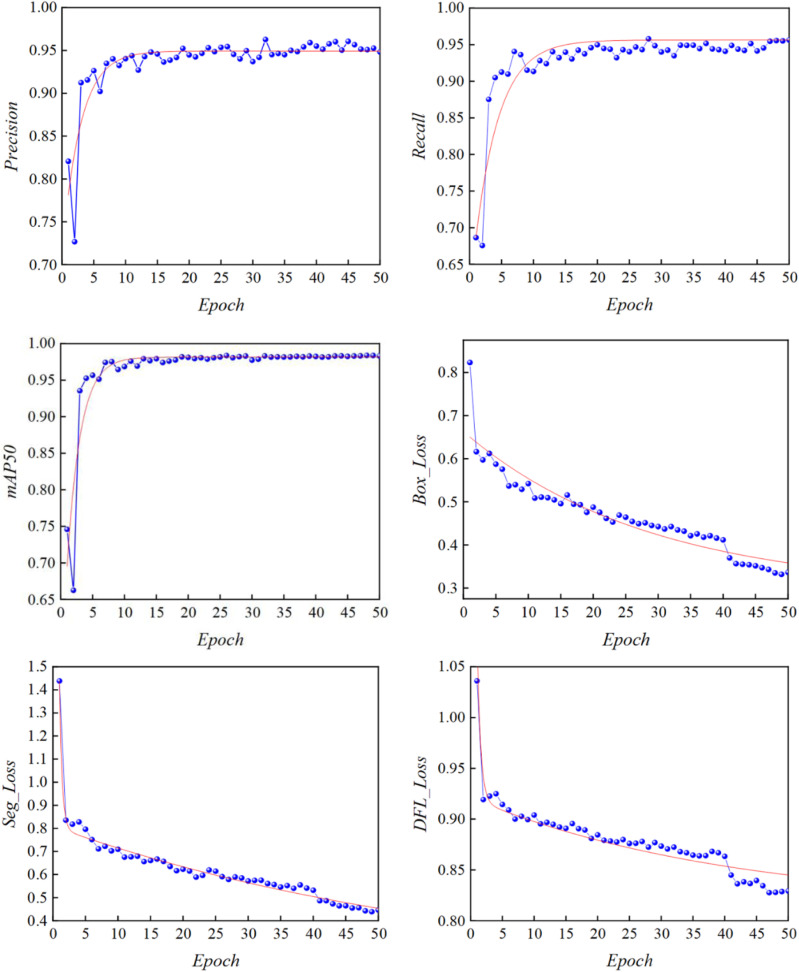
Image annotation results.

The relevant metrics Precision, Recall, mAP, Box_Loss, Seg_Loss, DFL_Loss of the improved YOLOv8 coal and gangue recognition model are shown in Fig 7.

#### Box_Loss.

YOLOv8 uses the GIoU_Loss loss function as the loss for prediction, Box is the GIoU loss function mean, the smaller its value the more accurate the prediction;

#### Seg_loss.

the detection loss mean, the smaller its value the more accurate the tar-get detection;

#### DFL_loss.

the free deformation loss, which is used to penalize the free deformation between the predicted frame and the real frame to improve the accuracy of the day scale detection, the smaller its value the more accurate the classification.

As can be seen from Fig 7, with the increase in the number of iterations, the three kinds of losses are decreasing, indicating that the model accuracy is gradually im-proved, and the model does not appear overfitting phenomenon, the final detection accuracy, recall rate are better performance, the average precision mean value is 96.88%.

### 4.4 Ablation experiments

In order to verify the performance effects on the YOLOv8 model by replacing and adding modules, module ablation experiments were designed, and the results of the ablation experiments are shown in Table 1.In the different models, Model A is the YOLOv8 model; Model B is the model after replacing the C2f module in the YOLOv8 backbone network with the DSC module; Model C is the model after adding the CBAM attentional mechanism module to the neck of YOLOv8; Model D is the model after re-placing the C2f module in the YOLOv8 backbone network with the DSC module and also after adding the CBAM attention mechanism module to the YOLOv8 neck.

**Table 1:** Results of ablation experiments.

Model	*Precision/%*	*Recall/%*	*mAP/%*	*FPS*
A	91.07	88.62	92.23	23.28
B	91.14	88.43	92.71	35.18
C	92.49	89.76	93.11	22.76
D	95.67	95.74	96.88	32.11

(1) Comparing model B and model A, it can be seen that replacing the C2f module in the YOLOv8 backbone network with the DSC module aims to reduce the model computation and improve the speed of the model processing images, and the experimental results show that replacing the DSC module can significantly reduce the model computation, and the number of images processed per second in model B is improved by 11.9 images per second compared with that in model A. At the same time, the detection precision and the recall rate do not have any big difference due to the reduction of the size.
(2) Comparing Model C and Model A, it can be seen that the CBAM attention mechanism module is added to the neck of YOLOv8 with the aim of enhancing the model’s recognition of small target images as a way to improve the overall recognition accuracy of the model, and the experimental results show that: the model’s precision rate is increased from 91.07% to 92.49% after the addition of the CBAM module, and the recall rate is increased from 88.62% to 89.76%. The average precision mean is in-creased from 92.23% to 93.11%, which significantly improves the accuracy of the mod-el, while the speed of image processing changes less.
(3) Comparing model D with model A, after introducing both DSC module and CBAM module, the precision rate of the model is improved from 91.07% to 95.67%, the recall rate is improved from 88.62% to 95.74%, the average precision mean is improved from 92.23% to 96.88%, the number of images processed per second is improved by 8.83, and the improved model has a significant improvement in both the recognition precision and recognition speed are significantly improved.

### 4.5 Comparison experiments

In order to objectively evaluate the recognition performance of the improved YOLOv8 on coal and gangue recognition images proposed in this paper, we select the more widely used YOLOv3, YOLOv5, YOLOv7, YOLOv8 four kinds of recognition models in the industry as a comparison under the same dataset and experimental equipment, and we select the number of parameters, the number of floating-point operations, the number of frames transmitted per second, and the average precision mean value as evaluation indicators for comparison experiments.

The experimental results are shown in Table 2.

**Table 2:** Comparison of detection results of different models.

Model	*number of participants/M*	*number of operations/10* ^*9*^	*FPS*	*mAP*
YOLOv3	62.51	68.76	26.74	81.13
YOLOv5	50.32	109.48	16.17	86.36
YOLOv7	39.65	105.24	15.18	88.54
YOLOv8	34.22	118.76	23.28	92.23
this paper	31.68	58.73	32.11	96.88

A comprehensive comparison of the six models is made in terms of model complexity, detection accuracy and detection speed.

(1) Model complexity

Model complexity is judged by the number of parameters indicator, i.e., the size of the weight file of the model. As can be seen from Table 2, the improved YOLOv8 model adopts a more complex network structure, and the number of parameters is 31.68M, which is the smallest among the 6 algorithms, indicating that the model size is small.

(2) Detection accuracy

Detection accuracy is judged by the mAP index, from the data in Table 2, it can be seen that the improved YOLOv8 model has a high accuracy of 96.88% in coal gangue image recognition, which is the maximum value among the six models, and it is 8.34% and 4.65% higher than that of YOLOv7 and YOLOv8 respectively, which indicates that the improved YOLOv8 model has a significant advantage in terms of accuracy.

(3) Detection speed

Detection speed is judged by the FPS index, from the data in Table 2, it can be seen that the improved YOLOv8 model processes 32.11 image frames per second, which is significantly higher than the other five models, and the image detection speed is fast.

**Fig 8:**
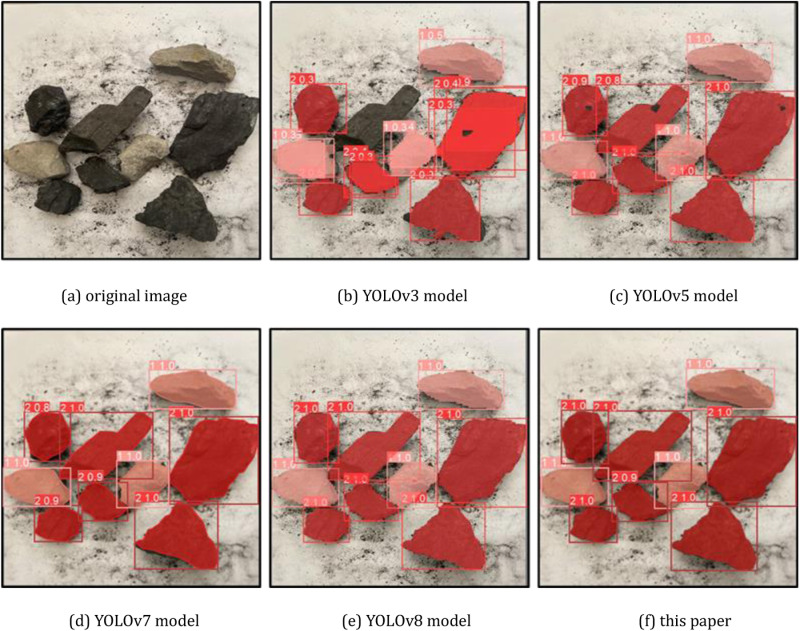
Comparison of partial detection effect of different models (case 1). (a) original image (b) YOLOv3 model (c) YOLOv5 model (d) YOLOv7 model (e) YOLOv8 model (f) this paper.

**Fig 9:**
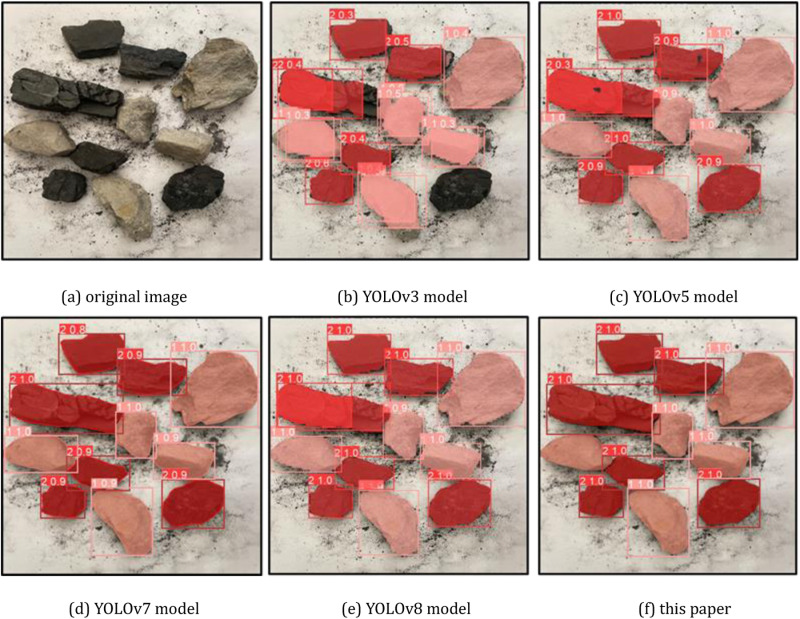
Comparison of partial detection effect of different models (case 2). (a) original image (b) YOLOv3 model (c) YOLOv5 model (d) YOLOv7 model (e) YOLOv8 model (f) this paper.

**Fig 10:**
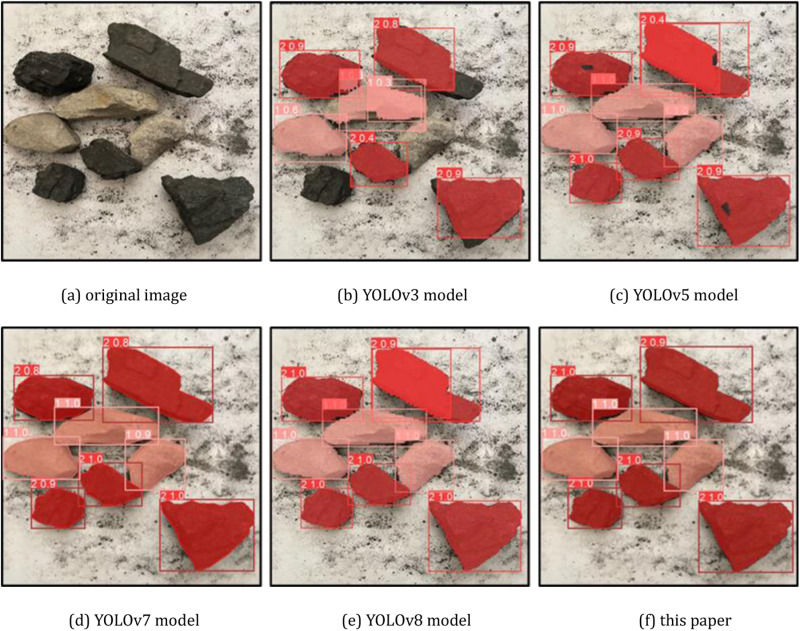
Comparison of partial detection effect of different models (case 3). (a) original image (b) YOLOv3 model (c) YOLOv5 model (d) YOLOv7 model (e) YOLOv8 model (f) this paper.

**Fig 11:**
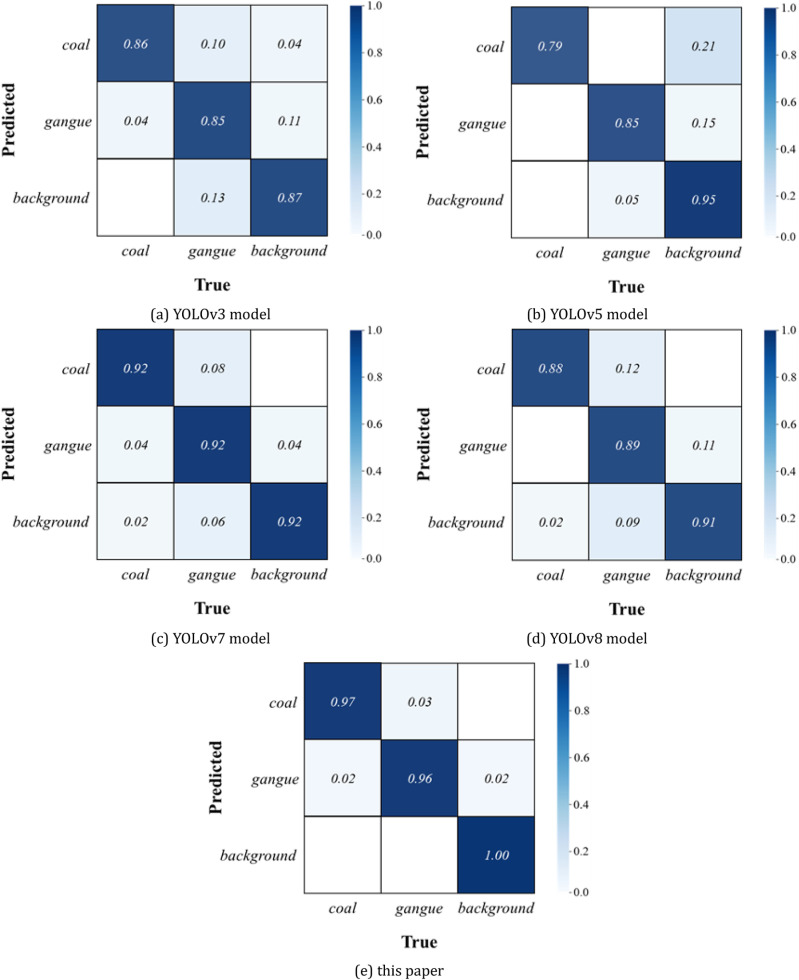
Confusion matrix of different models. (a) YOLOv3 model (b) YOLOv5 model (c) YOLOv7 model (d) YOLOv8 model (e) this paper.

Select a representative part of the detection results for visual comparison, the coal gangue image for segmentation detection, where the coal is dark red, gangue is light red, as shown in Fig 8, Fig 9 and Fig 10, and confusion matrix results are shown in Fig 11.

From the recognition results, the improved YOLOv8 model can accurately classify and identify the coal research and location detection, while the other models have different degrees of omission, from the recognition accuracy YOLOv8 model recognizes the coal with the highest confidence level among all the compared models.

## 5. Underground coal mine application effect

### 5.1 Transfer learning

The real coal mine underground environment is very complex and variable, low illumination, high dust, this condition directly using the model to identify the coal gangue image, the recognition accuracy obtained will inevitably decline. In the application of coal gangue recognition under complex conditions, it is necessary to first de-fog the collected images to enhance the distinction between coal and gangue, so as to facilitate the subsequent detection and recognition of the model. In this study, we only consider the recognition of coal gangue in a simple environment, and we do not consider the impact of illumination and dust on the recognition effect, which will be carried out by the authors in the subsequent research.

In order to enhance the generalization ability of the coal research segmentation model, the idea of migration learning is used to ensure that the model can accurately identify coal research in different working scenarios by using migration training based on the coal mine underground dataset.

The migration training in this paper adopts the coal research dataset of the underground working face, and the feature weights learnt from the migration training on the homemade coal research dataset in the laboratory are used as the initial weights of the model for training to simulate the segmentation scene of the coal gangue image in the low illumination and high dust environment.

### 5.2 Application effect

**Fig 12:**
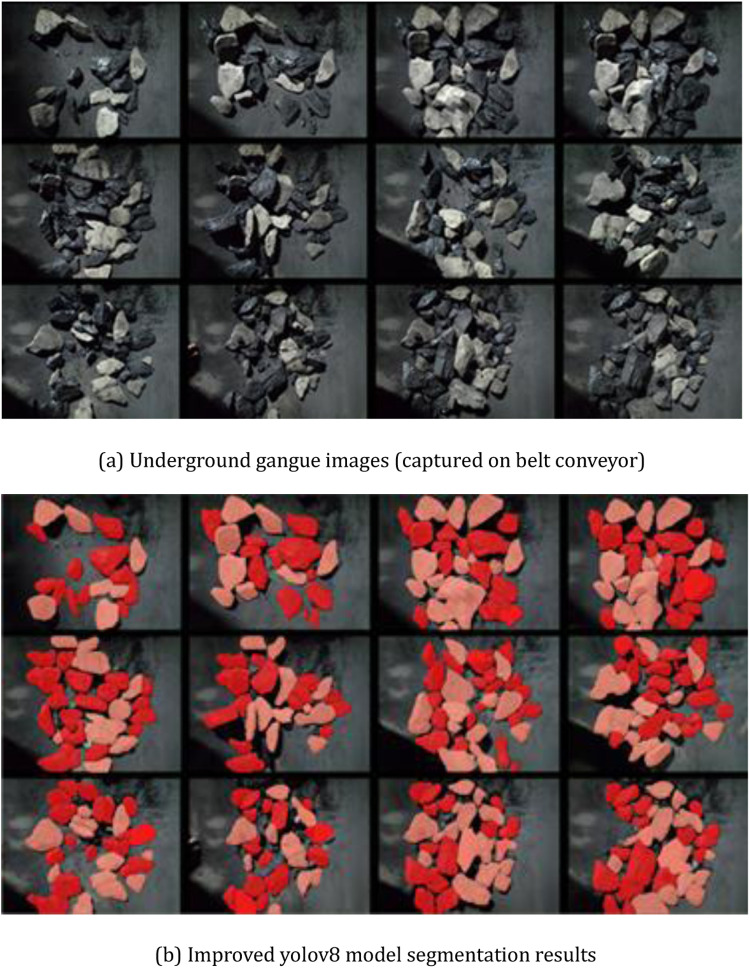
Segmentation results of coal gangue image on working face belt conveyor. (a) Underground gangue images (captured on belt conveyor) (b) Improved yolov8 model segmentation results.

**Fig 13:**
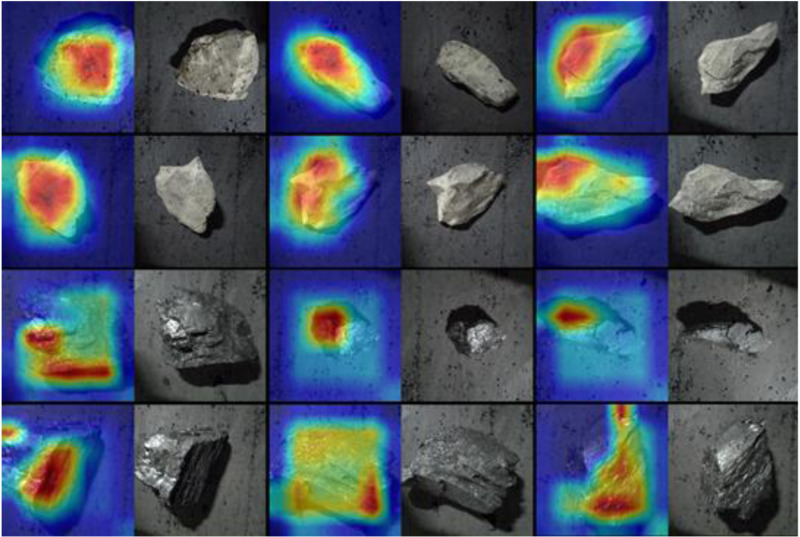
Heat map rendering of coal and gangue location.

In order to verify the feasibility of the improved YOLOv8 model proposed in this paper for practical application in the working face of underground coal mines, 1000 gangue images were collected from the belt conveyor of a coal mine and expanded by data enhancement methods, and a data set containing 6745 coal and gangue images was finally formed, and the training and test sets were divided according to the ratio of 8:2. From the test set, some randomly selected coal and gangue images are segment-ed, and the results are shown in Fig 12, the location of the coal gangue is rendered by the heat map shown in Fig 13, and it can be seen from the results that the improved YOLOv8 model has a very good global sensing ability, and can accurately recognize the coal features and carry out the annotation of the location and confidence level.

## 6. Conclusions

a. In this paper, an improved YOLOv8 coal and gangue recognition method for small targets is proposed. By replacing the standard convolution in part of the C2f module with depthwise separable convolution in the backbone network part of YOLOv8, it reduces the amount of model computation, and the method of adding the attention mechanism module in the neck to increase the model’s attention to the small target is improved, which enhances the speed of image detection and detection accuracy.
b. The data expansion of the original image was carried out by image rotation, decreasing brightness, increasing brightness, removing noise, and increasing noise, and the results of ablation experiments showed that after replacing the DSC module and introducing the CBAM module, the model precision was improved by 5.6%, and the recall rate was improved by 7.12%.
c. The image recognition results show that the size of the improved YOLOv8 model is 37.68M, the number of floating-point operations is 58.73 × 109 times, the FPS increases to 32.11 frames/s, and the average precision mean reaches 96.88%, which is higher than the YOLOv8 model in both detection speed and precision.
d. Using the transfer learning algorithm, the coal and gangue training model in the laboratory was applied to the underground coal mine recognition, and the improvement of the YOLOV8 model has a good gangue segmentation effect, and the location of the coal and gangue is determined accurately, which verifies the feasibility of the ap-plication of the model, and it provides an effective technological means for the coal gangue segmentation technology.

**Fig 14:**
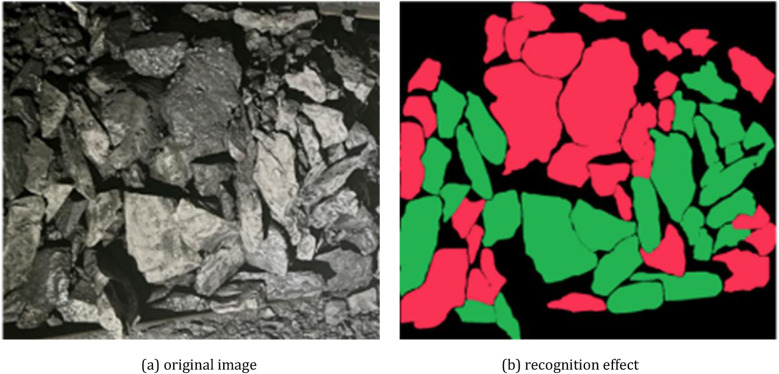
Recognition effect in case of mixed coal and gangue accumulation. (a) original image (b) recognition effect.

Future work

a. This research focuses on improving the YOLOv8 model, the acquired images are based on a simple environment and do not consider the recognition accuracy under the influence of illumination and dust, we recommend scholars to study the enhancement techniques of coal gangue images under different illumination and dust concentration conditions.
b. In this study, only the effect of separating coal and gangue is considered, the case of a large number of overlapping coal and gangue is not considered, and the authors do not have enough datasets for testing, so only an image collected at the coal mine working face is recognized using the existing model, as shown in Fig 14, which shows that the model accurately recognizes coal and gangue. Therefore, in future work, we will focus on coal and gangue images under complex conditions.

## Supporting information

S1 Data XXXXX. (RAR)
